# Induction and Characterization of Tetraploid Through Zygotic Chromosome Doubling in *Eucalyptus urophylla*

**DOI:** 10.3389/fpls.2022.870698

**Published:** 2022-04-27

**Authors:** Zhao Liu, Jianzhong Wang, Bingfa Qiu, Zhongcai Ma, Te Lu, Xiangyang Kang, Jun Yang

**Affiliations:** ^1^National Engineering Research Center of Tree Breeding and Ecological Restoration, College of Biological Sciences and Technology, Beijing Forestry University, Beijing, China; ^2^Key Laboratory of Genetics and Breeding in Forest Trees and Ornamental Plants, Ministry of Education, Beijing Forestry University, Beijing, China; ^3^The Tree and Ornamental Plant Breeding and Biotechnology Laboratory, National Forestry and Grassland Administration, Beijing Forestry University, Beijing, China; ^4^Guangxi Dongmen Forest Farm, Chongzuo, China; ^5^Science and Technology Section, Chifeng Research Institute of Forestry Science, Chifeng, China

**Keywords:** breeding, artificial polyploidy, high-temperature treatment, zygotic development period, trait variation, molecular mechanism

## Abstract

Improvements in plant growth can bring great benefits to the forest industry. *Eucalyptus urophylla* is an important plantation species worldwide, and given that ploidy increases are often associated with plant phenotype changes, it was reasoned that its polyploidization may have good prospects and great significance toward its cultivation. In this study, the zygotic development period of *E. urophylla* was observed through paraffin sections, and a correlation between the development time of flower buds after pollination and the zygotic development period was established. On this basis, it was determined that the 25th day after pollination was the appropriate time for a high temperature to induce zygotic chromosome doubling. Then tetraploid *E. urophylla* was successfully obtained for the first time through zygotic chromosome doubling induced by high temperature, and the appropriate conditions were treating flower branches at 44°C for 6 h. The characterization of tetraploid *E. urophylla* was performed. Chromosome duplication brought about slower growing trees with thicker leaves, larger cells, higher net photosynthetic rates, and a higher content of certain secondary metabolites. Additionally, the molecular mechanisms for the variation in the tetraploid’s characteristics were studied. The qRT-PCR results showed that genes mediating the tetraploid characteristics showed the same change trend as those of the characteristics, which verified that tetraploid trait variation was mainly caused by gene expression changes. Furthermore, although the tetraploid had no growth advantage compared with the diploid, it can provide important germplasm resources for future breeding, especially for the creation of triploids.

## Introduction

Improvements in plant growth are the main goals of tree breeders, and polyploidy has long been recognized as a major force in angiosperm evolution (Jiao et al., 2011). The evolution of most plants has involved one or more episodes of polyploidy (Soltis et al., 2009). However, the frequency of naturally occurring polyploidy is very low, which means it cannot meet the demand for tree growth improvements (Zhu et al., 1998). Nowadays, artificially induced polyploidy has been widely used for the generation and creation of new plant varieties (Lu et al., 2013; Guo et al., 2017). Compared with diploid plants, polyploidy plants usually grow robustly and have enlarged organs such as flowers, leaves, and fruits (Liu et al., 2007; Zhang et al., 2019). After polyploidization, plants often exhibit broader environmental adaptability, such as enhanced abiotic stress tolerance, and show significant increases in their potentially valuable secondary metabolite contents (Bon et al., 2020; Zhang et al., 2020). *Eucalyptus*, *Populus*, and *Salix* are fast-growing and important plantation species worldwide. *Eucalyptus* can provide a large amount of wood for industrial production. Its leaves contain oil that can be used as an effective pesticide or herbicide component (Hung et al., 2014; Dhakad et al., 2018; Stikhareva et al., 2021). Therefore, *Eucalyptus* polyploidization has good prospects and significance toward its breeding.

Although no naturally occurring *Eucalyptus* polyploids have been found, artificial mutagenesis could give rise to them (Guo et al., 2017). In previous studies on *Eucalyptus* tetraploid induction, only the use of colchicine to induce somatic chromosome doubling to obtain autotetraploids was reported, despite often obtaining a large number of mixoploids (Fernando et al., 2019; Silva et al., 2019). Compared with inducing the chromosome doubling of somatic cells, the mutagenesis approach to obtain tetraploids by treating zygotic cells after fertilization clearly shows great advantages (Lu et al., 2013; Guo et al., 2017). This is the case because tetraploids obtained by inducing chromosome doubling of zygotes develop from a single cell, and mixoploids will not be produced during the mutagenesis treatment period (Lu et al., 2013). Moreover, employing zygotes as mutagenic objects can also harness the hybrid effect produced by sexual mating design and obtain more genetic gains (Wang J. et al., 2010). Therefore, zygotic chromosome doubling induction is an important method to artificially induce tetraploidy. Colchicine, high temperature, or N_2_O are often used to block normal zygote development and promote chromosome doubling to obtain tetraploids in *Populus* and other tree species (Atwood, 1936; Wang J. et al., 2010; Gordej et al., 2019). As a physical mutagenic factor, high temperature is an efficient and environmentally friendly polyploid induction method in several hardwood tree species (Li et al., 2019; Yao et al., 2020). However, there has been no report on the induction of zygotic chromosome doubling by high temperature in *Eucalyptus*.

Tetraploids have been induced successfully in many plants, but there are few reports describing the tetraploids obtained by zygotic chromosome doubling. In studies of maize, rye, and other plants, tetraploids were successfully obtained by zygotic chromosome doubling after fertilization, but the characterization of tetraploids was not performed (Kato and Birchler, 2006; Gordej et al., 2019). Tetraploids have been obtained in *Populus*, a model tree species, through somatic or zygotic chromosome doubling (Lu et al., 2013; Wu et al., 2020). Although there have been characterizations of the tetraploid obtained by somatic chromosome doubling, the tetraploid obtained by zygotic chromosome doubling is unclear (Ren et al., 2021). In *Eucalyptus*, it was reported that the growth of tree height and diameter at breast height of autotetraploids obtained by somatic chromosome doubling was slower than that of diploids, and their content of secondary metabolites such as essential oils showed no significant change compared with that of diploids (Fernando et al., 2019). However, whether the variation trend in tetraploids obtained by zygotic doubling is consistent with that in those obtained by somatic doubling is the most important issue in breeding research and industrial production and applications.

This study carried out high-temperature-induced zygotic chromosome doubling to induce *Eucalyptus urophylla* tetraploids for the first time and explored the appropriate treatment time and conditions for inducing zygotic chromosome doubling. The characteristics of the tetraploids obtained by zygotic chromosome doubling were studied. Additionally, the molecular mechanism for the variation of characteristics after polyploidization of *E. urophylla* was also studied. This study provides a new path for *Eucalyptus* germplasm innovation and a reference for the development of *Eucalyptus* polyploid breeding in the future.

## Materials and Methods

### Plant Materials

*Eucalyptus urophylla* S. T. Blake used in this study are all from the same clone composed of 20 individual ramets. This clone was planted in a seed orchard at the Guangxi Dongmen Forest Farm (Guangxi Zhuang Autonomous Region, China) in 2015. After years of observation, the growth and the number of flowers within this clone were confirmed to be relatively consistent.

### Controlled Pollination

The flower branches of *E. urophylla* with a large number of flowers were selected as alternative flower branches for experiments. Controlled pollination was carried out after observing that most operculums changed from green to yellow. Pollination was performed following the method described by Assis et al. (2005). The pollen from other *Eucalyptus* clones collected in the previous year was used for controlled pollination. After pollination, the flower branches were bagged to exclude external pollen sources, and the bags were released 2 weeks after pollination.

### Observation of the Zygotic Development Process

The time of artificial pollination, when a large number of bud operculums of *E. urophylla* turned yellow, was set as 0. The flower buds were sampled every 24 h after pollination and then immediately placed inside a centrifuge tube filled with Carnoy’s fixative solution (acetic acid:ethanol, 1:3) (Tjio and Whang, 1962).

To observe mitosis in zygotes, the preserved flower buds were studied by the improved paraffin section technique described by Guo et al. (2019). Sections were observed and photographed under a microscope (BX51; Olympus) equipped with a CCD camera (DP70; Olympus).

### Zygotic Chromosome Doubling Using High Temperature

According to previous research, after correlating the development time of flower buds after pollination with the development period of zygotes, flower buds with different development times after pollination were treated at 40, 44, and 48°C for 3 and 6 h using the high-temperature treatment machine (Chinese invention patent No. ZL200610113448.X). Six replicate groups for each duration at each temperature were treated, and the untreated flower buds were used as a control. After treatment, the groups were marked according to treatment temperature and duration.

### Detection of Ploidy Levels

Capsules were harvested individually at maturity. Seeds were extracted and separated from the chaff by hand to permit their counting. Collected seeds were sown in the soil, and when the seedlings grew to approximately 20 cm, both flow cytometry and somatic chromosome counting were used to detect their ploidy levels according to the method of Yang et al. (2018).

### Measurement of Growth Traits of Tetraploids and Diploids in *E. urophylla*

After ploidy detection, diploids from full-sib families under the same treatment conditions were selected as controls, and tetraploids and diploids underwent clonal propagation. For tetraploid and diploid clones, 10 ramets were selected for biological replicates. When the plants had grown for 6 months, their height was measured with a tape, and the ground diameter was measured with a vernier caliper. All the leaves of each ramet of the two ploidy clones were counted, and five fully expanded mature leaves of two ploidy clones were randomly selected to measure the leaf length and width and the petiole length. The leaf area was recorded by a CI-203 handheld laser leaf area meter (Li-Cor Inc., Lincoln, NE, United States). Immediately after the measurement, the fresh weight of the leaves was obtained, and after drying in the oven, the dry weight was obtained. All data were measured three times, and the average was recorded.

### Leaf Anatomical Structure Analysis

The fully expanded mature leaves of diploids and tetraploids were selected as samples. Leaves were cut into 0.25 cm^2^ blocks, and their middle parts (including the leaf vein) were selected for the paraffin sections to observe mesophyll cell characteristics. Sections were stained with 1% safranine solution for 1 h, then transferred to a 0.1% fast green solution for 1 min. Sections were observed and photographed under a microscope (BX51; Olympus) equipped with a CCD camera (DP70; Olympus).

### Measurement of Photosynthetic Parameters

The fully expanded mature leaves of diploids and tetraploids were selected to measure their photosynthetic parameters. Net photosynthetic rate (Pn), stomatal conductance (Gs), intercellular carbon dioxide concentration (Ci), and transpiration rate (Tr) were measured using an LI-6400-02B portable photosynthesis system (Li-Cor Inc., Lincoln, NE, United States) at 8–10 o’clock on a sunny day in October 2020. All photosynthetic parameters were measured under a photosynthetic photon flux density of 1,400 mol^–2^ s^–1^, at 60% of relative humidity, and with a CO_2_ concentration of 400 μmol mol^–1^ held using a CO_2_ injection system (Cha-um and Kirdmanee, 2010). The instantaneous water use efficiency (WUE_i_) was calculated as Pn divided by Tr. Additionally, the chlorophyll fluorescence parameter Fv/Fm, reflecting the maximal photochemical efficiency of photosystem II (PSII), was measured on mature leaves using an MD-500 chlorophyll fluorescence spectrometer (Yi Zong Qi Technology Co. Ltd., Beijing, China).

### Stomatal Analysis

The fully expanded mature leaves of diploids and tetraploids were selected for stomatal observation. The leaf epidermis was peeled off and placed on the slide. Stomatal characteristics and chloroplast numbers were observed using ultraviolet or bright-field illumination and photographed under a microscope equipped with a CCD camera (BX51 and DP70; Olympus).

### Transmission Electron Microscopy Observation of Chloroplasts

The chloroplasts in the fully expanded mature leaves of diploids and tetraploids were prepared using the method of Wang et al. (2021). Chloroplast ultrastructures were observed using JEM-1010 electron microscopy (JEM-1010, Jeol Ltd., Tokyo, Japan).

### Physiological Parameter Measurements

The fully expanded mature leaves of diploids and tetraploids were selected for physiological parameter measurements. For the determination of the catalase (CAT) activity, peroxidase (POD) activity, malondialdehyde (MDA) content, total protein content, and soluble sugar content, their corresponding assay kits (Catalog No. A007-1-1, A084-3-1, A003-1-2, A045-2-2, and A145-1-1, Nanjing Jiancheng Bioengineering Ins., Nanjing, China) were used according to the manufacturer’s protocols.

### Foliar Oil Glands Isolation

A 100-mm^2^ section was excised from the middle part of fully expanded mature leaves of diploids and tetraploids and digested in pectinase, as described in Goodger et al. (2010). The leaf cuticle and attached epidermal layers were readily removed following digestion, as described in Goodger and Woodrow (2011). Foliar oil glands were isolated from the remaining leaf tissues by disruption and sieving (Goodger and Woodrow, 2011). The oil glands and mature leaves were observed using ultraviolet and bright-field illumination, respectively, and photographed under a microscope equipped with a CCD camera (BX51 and DP70; Olympus).

### qRT-PCR

The total RNA was isolated from the young, mature, and senescent leaves of *E. urophylla* using an RNAprep Pure Plant Plus Kit (Tiangen, China, cat DP441). Approximately 1.5 μg of RNA was reverse-transcribed into first-stand cDNA using a cDNA synthesis kit (Tiangen, China, cat KR106). The qRT-PCR reaction mixture consisted of 10 μl of 2 × TransStart^®^ Tip Green qPCR SuperMix (TransGen Biotech, China, cat AQ101), 0.6 μl of forward primer, 0.6 μl of reverse primer, 2 μl of cDNA template, 0.4 μl of 50 × ROX reference dye, and 6.4 μl of RNase-free ddH_2_O. qRT-PCR was performed on the Applied Biosystems 7500 Fast Real-Time PCR system for 39 cycles under the following conditions: 95°C for 15 min of pre-degeneration, 95°C for 10 s of degeneration, 58°C for 30 s of annealing, and 72°C for 30 s of extension. The samples were then heated to 95°C for 15 s and then 60°C for 1 min for dissolution curve analysis. Three technical replicates and three biological replicates were used for each sample and randomly selected gene. EuGADPH was a reference gene, and the primers used for qRT-PCR analysis are listed in Supplementary Table 1.

### Statistical Analysis

Measurements of leaf anatomical structure, oil glands, and stomatal parameters were obtained using the ImageJ software (version 1.51, NIH, Bethesda, MD, United States). The statistical analysis of tetraploids and diploids was performed using the SPSS version 19.0 software (SPSS Inc., Chicago, IL, United States). Significant differences among means were determined by Duncan’s multiple range tests at *P* ≤ 0.05.

## Results

### Correlation Between the Flower Bud Development and Zygotic Development Period

**FIGURE 1:**
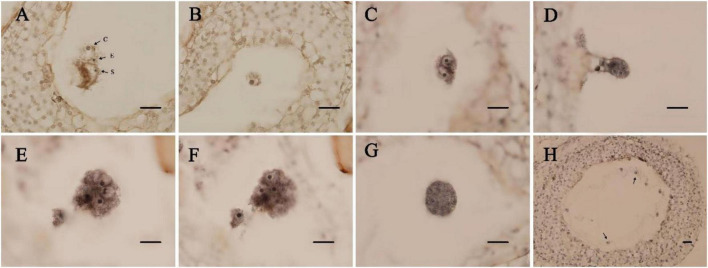
Paraffin section of ovules in *Eucalyptus urophylla.* **(A)** Central cell (c), egg cell (e), and persistent synergid (s); **(B)** zygote; **(C)** two-cell proembryo; **(D)** four-cell proembryo; **(E,F)** eight-cell proembryo; **(G)** multicellular embryo; and **(H)** free nuclear endosperm (bar = 200 μm).

We found a relationship between the development time of flower buds after pollination and the zygotic development period. According to the zygotic mitotic period, the development time of flower buds after pollination could be divided into 4 stages (Table 1). The double fertilization of *E. urophylla* was observed for the first time on the 7th day after pollination. At this time, central cells with fused polar nuclei, egg cells, and synergists were observed at the micropylar end of the embryo sac (Figure 1A). Subsequently, the process of double fertilization was completed, and dormant zygotes with obvious nucleoli could be observed in the embryo sac (Figure 1B). Zygotes experienced about 12 days of dormancy, and the first mitosis of zygotes was observed on the 23rd day after pollination. At this time, zygotes were divided to form two-cell proembryos (Figure 1C). In the next few days, fertilized eggs continued to develop, from four-cell proembryos to multicellular embryos (Figures 1D–G). The fertilized polar nucleus developed as the fertilized eggs developed. After multiple rounds of mitosis, multiple free nuclear endosperms scattered in the embryo sac could be observed (Figure 1H).

**TABLE 1:** Zygote development after pollination.

Time after pollination (day)	Development stages	Zygotic mitotic period
7–9	I	Double fertilization
10–22	II	Dormant zygote
23–25	III	Dormant zygote to four-cell proembryo
26–28	IV	Two-cell proembryo to multicellular embryo

### Inducing Zygotic Chromosome Doubling Through High Temperature

Based on the results from the cytological study of zygotic development (Figure 1), combined with the correlation between flower bud development time after pollination and the zygotic development period (Table 1), an experiment inducing zygotic chromosome doubling to obtain tetraploids by high temperature was carried out. Capsules were harvested at maturity the next year. In the treatment group with a temperature of 48°C and treatment durations of 3 or 6 h, the seeds could not be harvested, and the branches had dried up. However, the seeds could be harvested from groups treated with temperatures of 40°C or 44°C for 3 or 6 h (Table 2).

**TABLE 2:** *Eucalyptus urophylla* tetraploid production *via* zygote chromosome doubling by high temperature.

Time after pollination	Treatment temperature (°C)	Treatment duration (h)	No. of seeds	No. of Seedlings	No. of tetraploid	Tetraploid production rate (%)
23rd day	40	3	87	49	0	0
		6	56	37	0	0
	44	3	65	41	0	0
		6	52	36	0	0
	48	3	–	–	–	–
		6	–	–	–	–
24th day	40	3	97	42	0	0
		6	62	35	0	0
	44	3	58	30	1	3.33
		6	48	21	0	0
	48	3	–	–	–	–
		6	–	–	–	–
25th day	40	3	129	57	0	0
		6	97	47	0	0
	44	3	77	44	0	0
		6	69	31	2	6.45
	48	3	–	–	–	–
		6	–	–	–	–
26th day	40	3	154	68	0	0
		6	92	40	0	0
	44	3	78	25	0	0
		6	75	34	1	2.9
	48	3	–	–	–	–
		6	–	–	–	–
27th day	40	3	66	27	0	0
		6	19	9	0	0
	44	3	93	34	0	0
		6	145	47	0	0
	48	3	–	–	–	–
		6	–	–	–	–
28th day	40	3	87	49	0	0
		6	156	67	0	0
	44	3	236	130	0	0
		6	99	41	0	0
	48	3	–	–	–	–
		6	–	–	–	–

**FIGURE 2:**
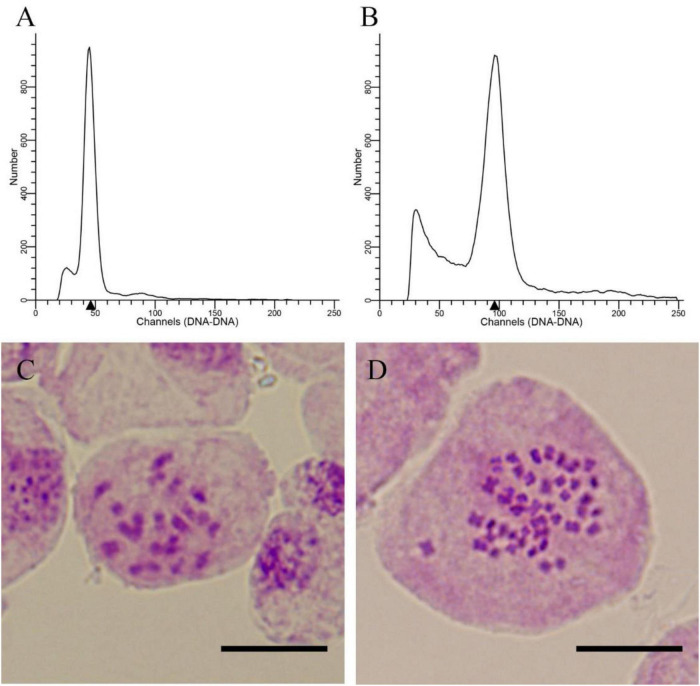
Ploidy level detection of *E. urophylla* offspring. **(A)** Histogram of the flow cytometry results for diploids; **(B)** histogram of the flow cytometry results for the tetraploid; **(C)** somatic chromosome counting for the diploid (2n = 2 × = 22); and **(D)** somatic chromosome counting for the tetraploid (2n = 4 × = 44).

The number of seeds and seedlings was counted. A total of 2,197 *E. urophylla* treated seeds were harvested, and 1,074 seedlings survived after sowing and transplantation into pots (Table 2). Using diploids as control, the ploidy levels of 1,074 seedlings were detected by flow cytometry, and a total of 4 tetraploids were detected. Then, the ploidy of 4 plants was further determined by somatic chromosome counting. The chromosome numbers of diploid *E. urophylla* are 2n = 2 × = 22, and the chromosome numbers of the 4 plants are 2n = 4 × = 44 (Figure 2), so they could be determined to be tetraploids. At the same time, the untreated seeds of *E. urophylla* were harvested as control groups, and no tetraploids were detected in the control groups. The results showed that tetraploids could be obtained from 24 to 26 days after pollination and treated at 44°C for 3 or 6 h. The appropriate conditions for inducing zygotic chromosome doubling of *E. urophylla* were treating flower branches at 44°C for 6 h on the 25th day after pollination.

### Comparison of Growth Traits

**FIGURE 3:**
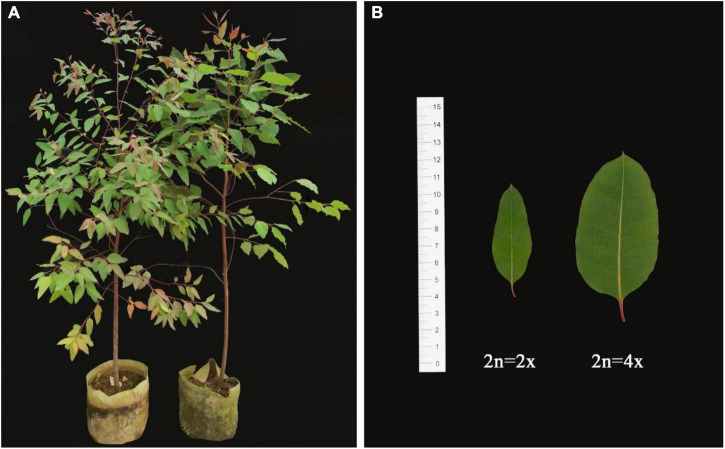
Morphological features of diploid and tetraploid plants in *E. urophylla.* **(A)** Clonal propagation ramet of diploids (left) and tetraploids (right); and **(B)** fully expanded leaf blades of diploids (left) and tetraploids (right).

Tetraploids and their diploid controls after undergoing clonal propagation were used to measure growth traits (Figure 3 and Table 3). When *E. urophylla* clones grew for 6 months, there was no significant difference in plant height between the tetraploids and diploids, but the ground diameter of the tetraploids decreased significantly. The number of leaves per ramet of tetraploids was only approximately half that of diploids. Several leaf attributes also varied between diploids and tetraploids. The mean leaf length of tetraploids was 9.43 cm, an increase of 78.26% compared to that of diploids. The mean leaf width of tetraploids was 2.62 cm, which was about twice that of diploids. The petiole connecting the leaf and stem of tetraploids was also longer than that of diploids. Additionally, the leaf area of tetraploids was more than three times that of diploids. After measuring leaf parameters, the leaf’s fresh mass was weighed. The leaf fresh mass of tetraploids was 344.00% higher than that of diploids. After drying, the leaf dry mass of tetraploids increased by 263.64% compared with that of diploid leaves. Taking the ratio of leaf mass to leaf area, the leaf fresh mass per unit area of tetraploid leaves increased by 19.23% compared with that of diploids, but there were no significant differences in leaf dry mass per unit area between tetraploids and diploids.

**TABLE 3:** Growth characteristics of tetraploid and diploid *Eucalyptus urophylla*.

Characteristics	Diploid	Tetraploid	Difference (%)	Significance
Plant height (cm)	74.33 ± 3.54	75.14 ± 2.84	–	NS
Ground diameter (cm)	10.50 ± 0.72	8.42 ± 0.69	−19.81	***
No. of leaves per plant	368.80 ± 29.97	165.60 ± 17.34	−55.10	***
Leaf length (cm)	5.29 ± 0.28	9.43 ± 1.47	+ 78.26	***
Leaf width (cm)	2.62 ± 0.16	5.27 ± 0.67	+ 101.15	***
Petiole length (cm)	0.72 ± 0.06	1.03 ± 0.111	+ 43.06	***
Leaf area (cm^2^)	10.32 ± 1.17	35.61 ± 8.56	+ 245.06	***
Leaf fresh mass (g)	0.25 ± 0.012	1.11 ± 0.305	+ 344.00	***
Leaf dry mass (g)	0.11 ± 0.009	0.40 ± 0.111	+ 263.64	***
Leaf fresh mass per unit area (g cm^–2^)	0.026 ± 0.0013	0.031 ± 0.0015	+ 19.23	***
Leaf dry mass per unit area (g cm^–2^)	0.011 ± 0.0007	0.011 ± 0.0009	–	NS

***for P ≤ 0.001 and NS for P > 0.05.

### Observation of Leaf Anatomical Features

**FIGURE 4:**
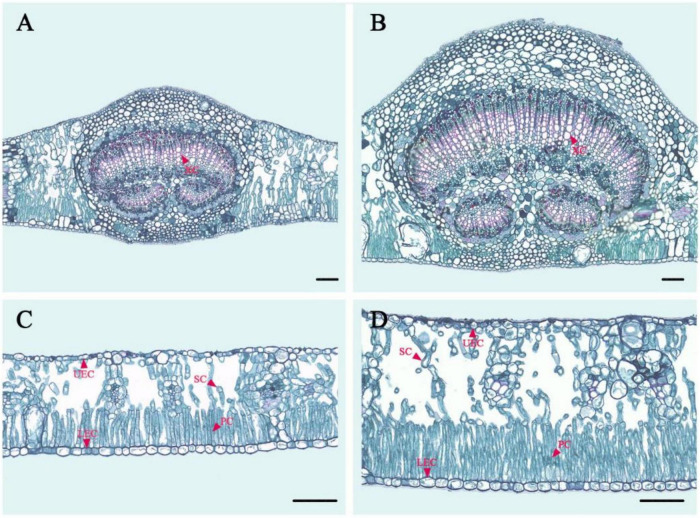
Paraffin section of a diploid and tetraploid leaf in *E. urophylla.* **(A)** Vein anatomical structure of the diploid; **(B)** vein anatomical structure of the tetraploid; **(C)** leaf anatomical structure of the diploid; and **(D)** leaf anatomical structure of the tetraploid. XC, xylem cells; UEC, upper epidermis cells; LEC, lower epidermis cells; SC, sponge cells; PC, palisade cells.

There were also differences in the leaf anatomical structure of tetraploids and diploids in *E. urophylla* (Figure 4 and Table 4). The leaves and veins of tetraploids were 67.40 and 129.06% thicker than those of diploids, respectively. The xylem, palisade, and sponge cells were significantly enlarged in tetraploids compared to diploids. Moreover, the palisade cell arrangement in tetraploid leaves was denser, with an average of 112 palisade cells per unit length (mm^–1^). In addition, the area of upper and lower epithelial cells in tetraploid leaves was significantly larger than that in diploid leaves.

**TABLE 4:** The leaf anatomical structure of diploid and tetraploid *Eucalyptus urophylla*.

Characteristics	Diploid	Tetraploid	Difference (%)	Significance
Leaf thickness (μm)	243.11 ± 6.39	406.97 ± 3.77	+67.4	***
Veins thickness (μm)	513.16 ± 2.01	1175.43 ± 5.94	+129.06	***
Palisade cells (μm)	88.50 ± 2.39	133.21 ± 2.23	+50.52	***
Sponge cells (μm)	31.86 ± 2.73	41.42 ± 3.76	+30.01	**
Density of palisade cells (mm^–1^)	82.67 ± 2.79	112.00 ± 3.80	+35.48	***
Xylem cells (μm^2^)	285.00 ± 18.47	694.00 ± 47.37	+143.51	***
Upper epidermis cells (μm^2^)	452.76 ± 33.93	690.01 ± 78.29	+52.4	***
Lower epidermis cells (μm^2^)	149.83 ± 60.97	299.83 ± 85.24	+100.11	***

***for P ≤ 0.001 and **for 0.001 < P ≤ 0.01.

### Measurement of Photosynthetic Characteristics

The measurement results of photosynthetic parameters of *E. urophylla* tetraploids and diploids are shown in Table 5. The net photosynthetic rate (Pn) of tetraploids was 14.51 μmol m^–2^ s^–1^, significantly higher than that of diploids. The water use efficiency (WUE_i_) of tetraploids was also significantly higher than that of diploids. However, there were no significant differences between tetraploids and diploids in intercellular carbon dioxide concentration (Ci), stomatal conductance (Gs), and transpiration rate (Tr). The chlorophyll fluorescence parameters were also measured on the same day. There was no significant difference in the maximal photochemical efficiency of photosystem II (PSII) between tetraploids and diploids.

**TABLE 5:** Leaf photosynthetic parameters of diploid and tetraploid *Eucalyptus urophylla*.

Characteristics	Diploid	Tetraploid	Significance
Pn (μmol m^–2^ s^–1^)	12.53 ± 1.88	14.51 ± 1.16	*
Gs (mol m ^2^ s^–1^)	0.171 ± 0.048	0.172 ± 0.024	NS
Ci (μmol m^–1^)	248.09 ± 23.50	241.2 ± 19.54	NS
Tr (mmol m^–2^ s^–1^)	4.079 ± 1.130	3.604 ± 0.431	NS
WUE_i_	3.26 ± 0.85	4.08 ± 0.59	*
Fv/Fm	0.644 ± 0.037	0.627 ± 0.035	NS

*for 0.01 < P ≤ 0.05 and NS for P > 0.05.

### Observation of Stomata and Chloroplast Characteristics

**FIGURE 5:**
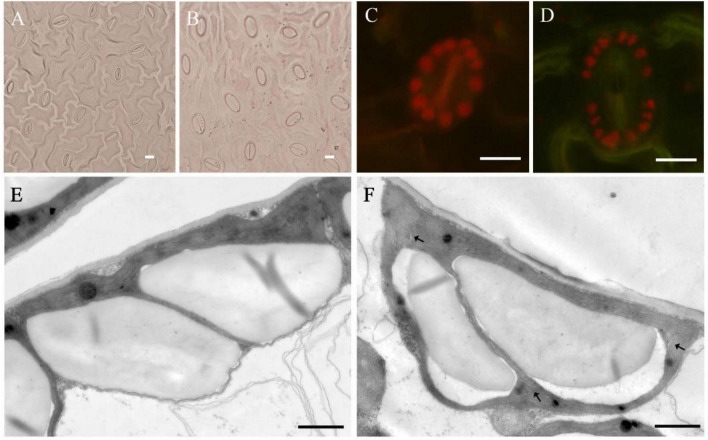
Stomatal and chloroplast characteristics of tetraploids and diploids in *E. urophylla.* Stomata in the leaf of diploid **(A)** and tetraploid **(B)**; chloroplast number in stomata of diploid **(C)** and tetraploid **(D)**; ultrastructure of mesophyll cell chloroplasts of the diploid **(E)**; and tetraploid **(F)**. Arrows indicate sparse stroma lamella (**A–D**, bar = 10 μm; **E,F**, bar = 1 μm).

There were significant differences in the number and density of stomata on the leaves between tetraploids and diploids (Figure 5 and Table 6). The stomatal size of tetraploids increased significantly, with a length of 20.16 μm and a width of 12.71 μm, which were 34.76 and 59.87% larger than that of diploids, respectively. However, while the stomata of tetraploids were enlarged, their stomatal density decreased by 22.67% compared with that of diploids, and there was only a mean of 360.75 stomata per mm^2^ of tetraploid leaves. The mean number of chloroplasts in the guard cells of tetraploids was 17.5, which was significantly higher than that of diploids. The transmission electron microscopy results of diploid and tetraploid chloroplasts showed that the starch grains in diploid chloroplasts were fuller. Osmiophilic granules were more widely distributed in tetraploid chloroplasts, and the matrix lamella started to become loose locally, which was a manifestation of senescence and damage.

**TABLE 6:** Stomatal characteristics of tetraploid and diploid *Eucalyptus urophylla*.

Ploidy	Stomatal length (μm)	Stomatal length (μm)	Stomatal density (No. mm^–2^)	No. of chloroplasts
Diploid	14.96 ± 1.19	7.95 ± 0.80	466.50 ± 23.13	11.13 ± 0.99
Tetraploid	20.16 ± 1.93	12.71 ± 0.95	360.75 ± 42.61	17.67 ± 1.35
Significance	***	***	***	***

***for P ≤ 0.001.

### Measurement of Physiological Characteristics

The leaf physiological parameter measurements of *E. urophylla* diploids and tetraploids are shown in Table 7. There were significant differences in the contents of MDA, POD, and soluble sugars between tetraploids and diploids. However, there were no differences in the content of total protein and CAT between tetraploids and diploids. POD and soluble sugar contents were significantly elevated in tetraploids, but the MDA content decreased by 36.24% compared with that in diploids.

**TABLE 7:** Physiological parameters of diploid and tetraploid *Eucalyptus urophylla*.

Ploidy	Protein (mg⋅gFW^–1^)	CAT (U⋅mgprot^–1^)	MDA (nmol⋅mgprot^–1^)	POD (U⋅mgprot^–1^)	Soluble sugar (mg⋅gFW^–1^)
Diploid	2.50 ± 0.51	325.67 ± 53.71	9.05 ± 0.86	11.61 ± 1.50	16.35 ± 0.79
Tetraploid	3.44 ± 0.49	344.57 ± 60.35	5.77 ± 0.80	16.71 ± 0.55	19.99 ± 0.83
Significance	NS	NS	***	***	**

***for P ≤ 0.001, **for 0.001 < P ≤ 0.01, and NS for P > 0.05.

### Observation of Oil Gland Characteristics

**FIGURE 6:**
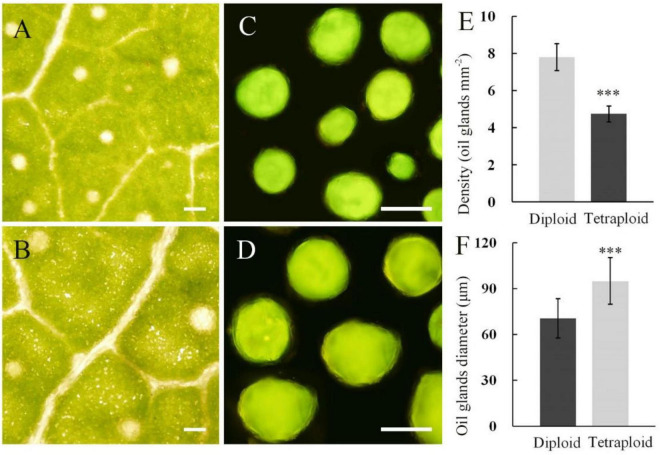
Oil gland characteristics of *E. urophylla* tetraploids and diploids. Oil glands in the leaf of diploid **(A)** and tetraploid **(B)**; oil glands isolated from the diploid **(C)** and tetraploid **(D)** leaf; **(E)** for measurements of oil gland density; and **(F)** for measurements of oil gland diameter (bar = 100 μm). ***for *P* ≤ 0.001.

Under the microscope, differences in oil gland diameter and density between diploid and tetraploid leaves were observed (Figure 6). The mean diameter of oil glands in the leaves of tetraploids was 94.81 μm, being significantly larger than that of diploids. However, there was only a mean of 4.75 oil glands per cm^2^ of tetraploid leaves, with a density that was significantly lower than that of diploid leaves.

### Molecular Mechanism of Trait Variation

**FIGURE 7:**
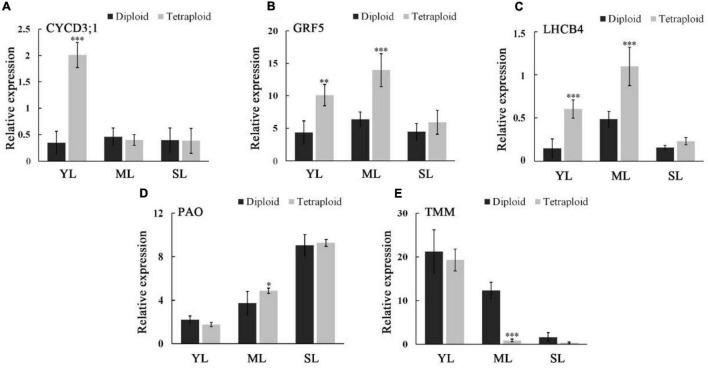
Expressions of *CYCD3;1* **(A)**, *GRF5* **(B)**, *LHCB4* **(C)**, *PAO1* **(D)**, and *TMM* **(E)** were measured by real-time qRT-PCR in young leaves (YM), mature leaves (ML), and senescent leaves (SL) of diploids and tetraploids. ***for *P* ≤ 0.001, **for 0.001 < *P* ≤ 0.01, and *for 0.01 < *P* ≤ 0.05.

To deeply understand the molecular mechanism of tetraploid trait variation, several genes related to leaf growth and photosynthesis were selected for qRT-PCR (Figure 7). Leaf growth is a dynamic process, and according to the development time, the leaves were divided into three stages: young, mature, and senescent leaves. The expression patterns of genes controlling different traits were approximately the same in the three developmental stages of diploid and tetraploid leaves. The expression levels of *CYCD3;1*, a gene related to cell division, were relatively stable in diploid leaves but upregulated significantly in young tetraploid leaves. The expression patterns of *GRF5*, related to leaf expansion, and *LHCB4*, related to photosynthesis, were the same in diploids and tetraploids, and their expression levels were the highest in mature leaves. Compared with diploids, *GRF5* and *LHCB4* showed a higher expression across different stages in tetraploid leaves, and significant expression differences between tetraploids and diploids appeared in young and mature leaves. The expression of *PAO* related to chlorophyll degradation was gradually upregulated with the change in leaf stages, and that in tetraploid mature leaves was significantly higher than that in diploid leaves. *TMM*, the gene related to stomatal formation, showed an expression pattern opposite to that of *PAO*. The expression of *TMM* in tetraploids was lower than that in diploids during the entire leaf growth process, and especially in mature leaves, there was a significant difference between them.

## Discussion

Most of the previous studies on tetraploids focused on somatic chromosome doubling in *Eucalyptus* (Silva et al., 2019; Castillo et al., 2020; Moura et al., 2020). Induced zygotic chromosome doubling has been successfully applied in several plants (Randolph, 1932; Atwood, 1936; Guo et al., 2017). Surprisingly, there have been no reports on zygotic chromosome doubling to obtain *Eucalyptus* tetraploids. In this study, four *E. urophylla* tetraploids were successfully obtained by high-temperature treatment, and no mixoploidy was found in the process of ploidy level detection. Compared with somatic chromosome doubling, the polyploids obtained by zygotic chromosome doubling are stable, and there is no need to screen for mixoploidy (Lu et al., 2013). At the same time, zygotic chromosome doubling is a method to obtain polyploids by sexual reproduction. If controlled pollination is carried out before high-temperature treatment, the method has the potential to obtain multi-genotype and multi-trait polyploids (Wang J. et al., 2010; Guo et al., 2017). Additionally, zygotic chromosome doubling was induced by high temperature without any chemical mutagenic agent to avoid the risk of environmental pollution. Therefore, zygotic chromosome doubling induced by high temperature is an option for *Eucalyptus* germplasm innovation.

In this study, we explored the induction of zygotic chromosome doubling in *Eucalyptus* by high temperatures and found some factors restricting the efficiency of zygotic chromosome doubling. Previous studies have shown that the essence of chromosome doubling induced by high temperature was that high temperatures could affect spindle formation in a specific period of cell division, thus blocking the normal cell division process, and then causing chromosome doubling (Kang et al., 2000; Li et al., 2019). Therefore, the key to the successful induction of zygotic chromosome doubling of *E. urophylla* lies in the appropriate timing of mutagenesis treatment (Lu et al., 2013). In the cytological observation of zygote development, ovules in a two-cell proembryo stage and multicellular embryo stage could be observed in the same flower bud. This phenomenon of asynchronous zygotic development is common in plants (Pound et al., 2002, 2003) and seriously affects the judgment of the appropriate period of zygotic division for high-temperature treatment. At the same time, the phenomenon of asynchronous zygotic development also reduces the probability of applying the high-temperature treatment during the appropriate period of zygotic division, which greatly affects the mutagenesis efficiency and tetraploid yield. Therefore, we proposed that improving the proportion of zygotes in the appropriate division period during mutagenesis treatment to increase the tetraploid induction efficiency will be the focus of further research.

Additionally, the treatment temperature was chosen for two main reasons. One reason is that *E. urophylla* always blooms in the summer (from June to August in southern China), and the daily extreme temperature reaches 40°C, but tetraploid offspring have not been found in nature or in the untreated control groups of this study, indicating that temperatures of 40°C or lower are insufficient to induce zygotic chromosome doubling in *E. urophylla*. Our study showed that seeds could not be harvested when the treatment temperature reached 48°C, so the suitable treatment temperature for the mutagenesis of *E. urophylla* should be between 40 and 48°C. To determine the appropriate treatment conditions for zygotic chromosome doubling, the process of zygotic development of *E. urophylla* was tracked, and the treatment was performed at different temperatures. Using the pollination time as the reference, the results showed that a treatment at 44°C for 6 h on the 25th day after pollination was the most appropriate for inducing zygotic chromosome doubling in *E. urophylla*. This conclusion provides an important reference for polyploid induction in other *Eucalyptus* species.

As is the case in other polyploids, the leaf characteristics of tetraploids obtained by zygotic chromosome doubling were significantly different from those of diploids. The results showed that the leaf area of tetraploids was significantly larger than that of diploids. The observation of leaf anatomical structures found that the cell size and leaf thickness of tetraploids were also significantly increased compared with those of diploids, which may be the reason for the increase in water content in tetraploid leaves, making the leaf fresh mass per unit area of tetraploids significantly higher than that of diploids. The increase in leaf area is a common phenomenon after polyploidization, such as that seen in tetraploid *Morus multicaulis* and *Platanus acerifolia*, which have larger leaves than diploids (Liu et al., 2007; Wang X. L. et al., 2010). However, it should be noted that the changes in leaf length and leaf width after polyploidization are not consistent. For example, Fernando et al. (2019) reported that the ratio of leaf length to leaf width of the autotetraploid *Eucalyptus polybractea* did not change compared with that of the diploid. In this study, the leaf width of tetraploids with zygotic chromosome doubling increased more than the leaf length, resulting in a change of leaf shape, which may be due to gene recombination in the process of sexual reproduction (Sattler et al., 2016). This showed that compared with the autotetraploid obtained by somatic chromosome doubling, tetraploids based on zygotic chromosome doubling have more abundant genetic variation, which is very important for breeding germplasm with high yield, good resistance, and other properties with high economic value.

The Pn of *E. urophylla* tetraploids was significantly higher than that of diploids. The Pn per unit leaf area is the product of the Pn of a single cell and the number of photosynthetic cells per unit leaf area (Warner and Edwards, 1989). If cell accumulation is changed to allow more cells per unit leaf area, the Pn per unit leaf area will increase (Warner and Edwards, 1993; Cao et al., 2018). This study found that the number of palisade cells per unit length increased significantly in the longitudinal section of tetraploid leaves. This may be the main reason for the increase in Pn of tetraploids. Additionally, the number of chloroplasts in the mesophyll cells of tetraploids is significantly higher than that of diploids, which may be another reason for the increase in Pn (Jellings and Leech, 1984; Warner et al., 1987). The enlargement of guard cells and stomatal volume caused by the polyploidization did not significantly change the Ci, Gs, and Tr of *E. urophylla*. This may be because the number of stomata per unit leaf area in tetraploids declined in proportion to the increase in stomatal size, and the parameters may be the same as in diploids, having smaller stomata but with higher stomatal density (Harrison et al., 2020). The chlorophyll fluorescence parameters of diploids did not change significantly, which was consistent with the study by Liao et al. (2016) in *Populus*. In general, chlorophyll fluorescence parameters are essentially constant in living plants unless they are exposed to extreme biological and abiotic stress conditions (Björkman and Demmig, 1987).

Growth characteristics of diploids and tetraploids were measured in *E. urophylla*, and tetraploids were found to grow more slowly. Although there was no difference in plant height between tetraploids and diploids, the basal diameter growth of tetraploids was significantly slower than that of diploids. In a previous study of *Eucalyptus polybractea*, Fernando et al. (2019) reported that tetraploids may be more susceptible to water stress affecting their growth. This study found that tetraploids with the same seedling age had fewer leaves. Leaves were the main organ for photosynthesis (Smith et al., 1997), and reductions in the number of leaves led to a decrease in the Pn of the whole plant and carbon fixation efficiency, which may be a reason for their slower growth (Offermann and Peterhansel, 2014). On the contrary, although the Pn of tetraploids increased, the Gs did not significantly change. This restricted the capacity for CO_2_ absorption and limited the upper limit of improvement of Pn (Harrison et al., 2020). However, the increase in mesophyll cell size and density increased the energy required to produce cells and maintain cell functions (Nowicka et al., 2018; Siqueira et al., 2018). The increase in carbon fixation caused by the Pn increase cannot compensate for the increase in carbon metabolism caused by changes in cell size and density, which may be another reason for the slow growth of tetraploids.

After polyploidization, the physiological parameters and secondary metabolites of *E. urophylla* also changed. The MDA content decreased and the POD content increased in tetraploid leaves. Due to these contents are related to the stress resistance of plants, it can be inferred that tetraploids may have stronger environmental adaptability (Zhou et al., 2020). Additionally, an increase in soluble sugar content was detected in tetraploids. This result was also reported in the study of apple and ginger polyploidy (Xue et al., 2017; Zhou et al., 2020). This may be caused by changes in cell volume or cell wall components after polyploidization (Madadi et al., 2021). At the same time, the size of oil glands in leaves also increased with polyploidization (Fernando et al., 2019). Although the interaction between the increase in oil gland volume and the decrease in density led to no significant changes in the content of essential oils after polyploidization in *Eucalyptus polybractea*, tetraploids containing larger oil glands are a potential resource for cultivating new *Eucalyptus* varieties with higher essential oil contents.

The changes in plant characteristics after polyploidization are mainly caused by changes in gene expression. In plants, genome replication increases the complexity of genetic composition and affects plant gene expression (Scarrow et al., 2021). After polyploidization, the expression of genes related to chlorophyll synthesis in *Arabidopsis thaliana* and *Chrysanthemum nankingense* was upregulated, resulting in enhanced photosynthesis (Ni et al., 2009; Dong et al., 2017). The upregulated expression of *GRF5* in *Populus* polyploids leads to its production of leaves with a larger leaf area (Wu et al., 2021). Exploring the differences in gene expression after plant polyploidization is of great significance for analyzing the variation of polyploid traits (Du et al., 2020). In this study, genes related to leaf growth and expansion were analyzed by qRT-PCR. *CYCD3;1* plays a role in cell division and proliferation, and it was only differentially expressed in diploid and tetraploid young leaves, which indicates that a significant increase in mesophyll cells of the tetraploid mainly occurred in young leaves (Dewitte et al., 2003; de Jager et al., 2009). In *Populus*, overexpressing *CYCD3;1* could significantly increase the number of cells in leaves, but cell volume decreased (Guan et al., 2021). However, the cell volume increased after tetraploidy in *E. urophylla* (Figures 4D, 6F). The upregulated expression of *GRF5* can increase cell volume (Wu et al., 2021), and the expression of *GRF5* in both young and mature tetraploid leaves was significantly higher than that in diploid leaves, which may cause the enlargement of cell volume. The increase in cell number and volume can remarkably increase plant organ size, which also suggests that changes in plant organs after polyploidy are caused by multiple gene expression changes.

This study also quantified the genes causing the changes in Pn of tetraploid *E. urophylla*. *PAO* encodes an enzyme that catalyzes chlorophyll decomposition, and its expression positively correlates with leaf senescence (Pružinská et al., 2003, 2005). The expression of *PAO* was only significantly increased in mature tetraploid leaves at the level of 0.01% < *P* < 0.05%. The transmission electron microscopy observation of chloroplasts also found only a slight difference between tetraploids and diploids, indicating that chlorophyll degradation may not be an important reason for the differences in the Pn of mature leaves of tetraploids and diploids. *LHCB4* encodes a protein involved in PSII composition (de Bianchi et al., 2011). The expression of *LHCB4* in both young and mature tetraploid leaves was significantly higher than that in diploid leaves. It has been speculated that the difference in the PSII formation process may be one of the reasons for the enhanced photosynthesis in tetraploids. Additionally, stomata also affect photosynthesis (Harrison et al., 2020). *TMM* is a key gene controlling stomatal development (Chowdhury et al., 2021), and the significant downregulation of its expression in tetraploids resulted in a decrease in leaf stomatal density, which restricted the upper limit of tetraploid photosynthesis. The above results provide a new perspective for understanding the changes in Pn of polyploid plants.

## Conclusion

In this study, tetraploid *E. urophylla* was successfully obtained for the first time by inducing zygotic chromosome doubling at high temperatures, and changes in its characteristics and gene expression were studied. Although the tetraploid grew slowly, it showed some advantages, such as larger and thicker leaves, higher Pn, and higher secondary metabolite contents. Trends in the expression of genes related to trait formation were the same as those of traits, indicating that tetraploid trait variation was mainly caused by changes in gene expression. Although the tetraploid had no growth advantages, it can provide important germplasm resources for future breeding, especially for the creation of triploids.

## Data Availability Statement

The datasets presented in this study can be found in online repositories. The names of the repository/repositories and accession number(s) can be found in the article/Supplementary Material.

## Author Contributions

JY and XK conceived and designed the research. ZL, JW, and ZM conducted the experiments. ZL, BQ, and TL collected and analyzed the data. ZL and JY wrote the manuscript. XK provided valuable suggestions on the manuscript. All authors read and approved the final manuscript.

## Conflict of Interest

The authors declare that the research was conducted in the absence of any commercial or financial relationships that could be construed as a potential conflict of interest.

## Publisher’s Note

All claims expressed in this article are solely those of the authors and do not necessarily represent those of their affiliated organizations, or those of the publisher, the editors and the reviewers. Any product that may be evaluated in this article, or claim that may be made by its manufacturer, is not guaranteed or endorsed by the publisher.
