# Supplementary figures and images for: Induction and Characterization of Tetraploid Through Zygotic Chromosome Doubling in Eucalyptus urophylla
Source: Front Plant Sci. 2022 Apr 27;13:870698. doi: 10.3389/fpls.2022.870698 (PMC9094141; doi:10.3389/fpls.2022.870698)

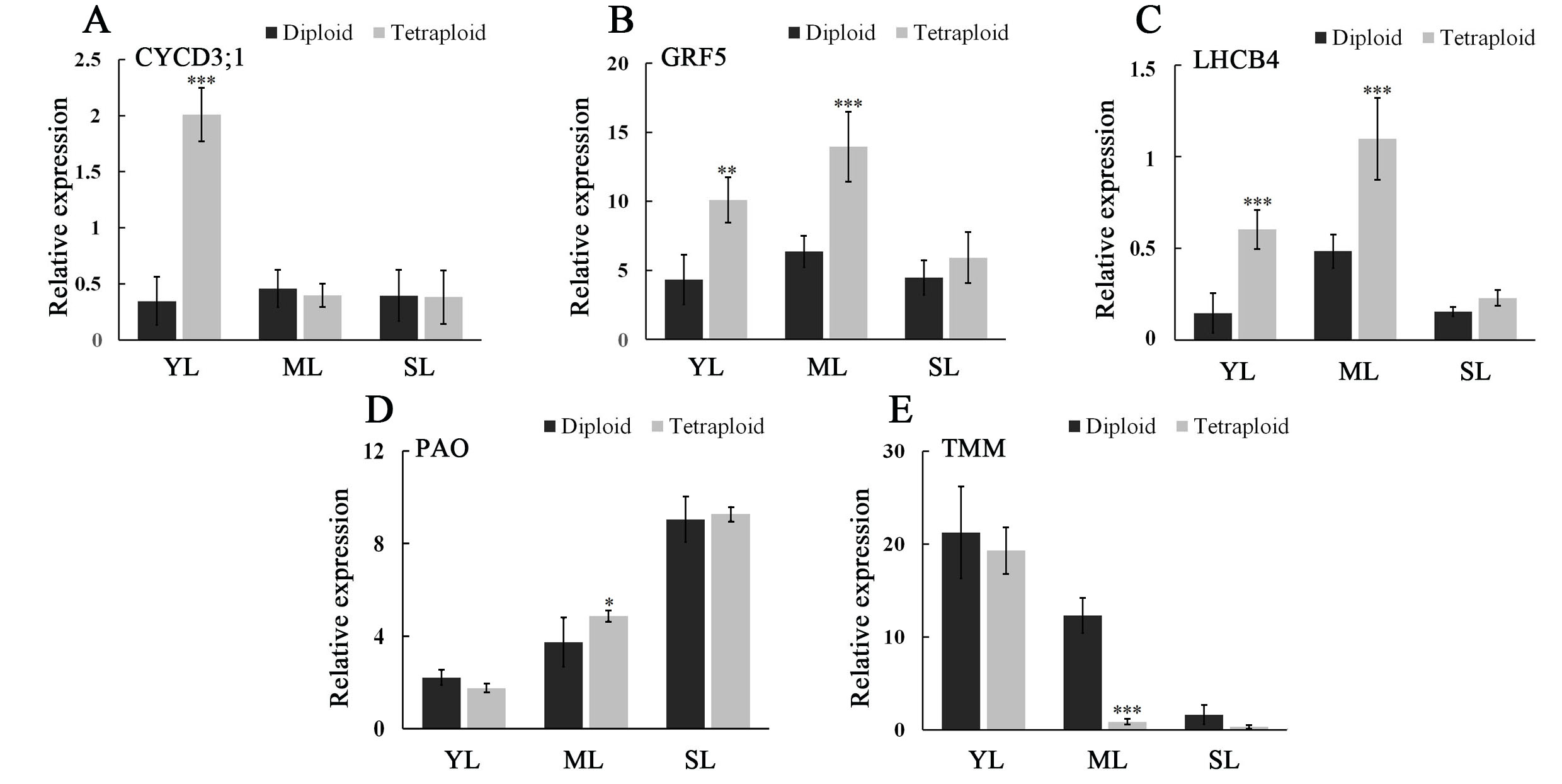

Supplement: Supplementary file 1 [file Data_Sheet_2.ZIP › Fig 7.jpg]

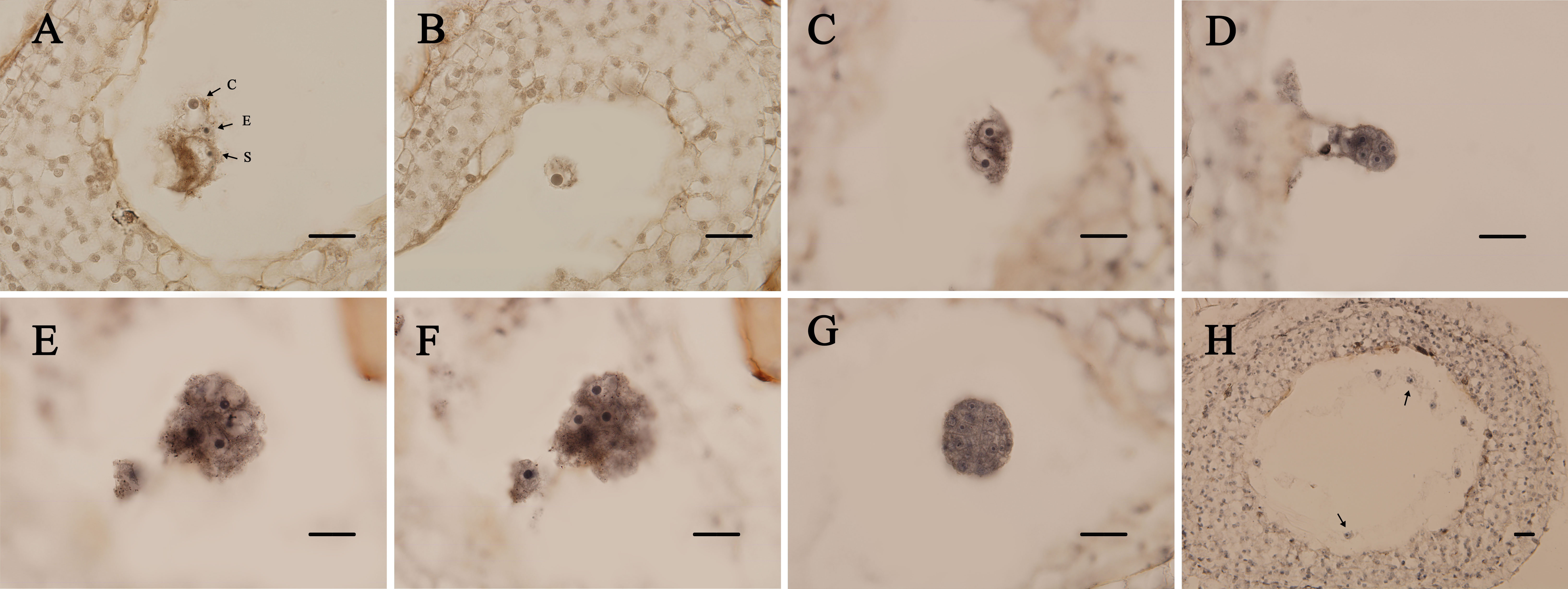

Supplement: Supplementary file 1 [file Data_Sheet_2.ZIP › Fig 1.jpg]

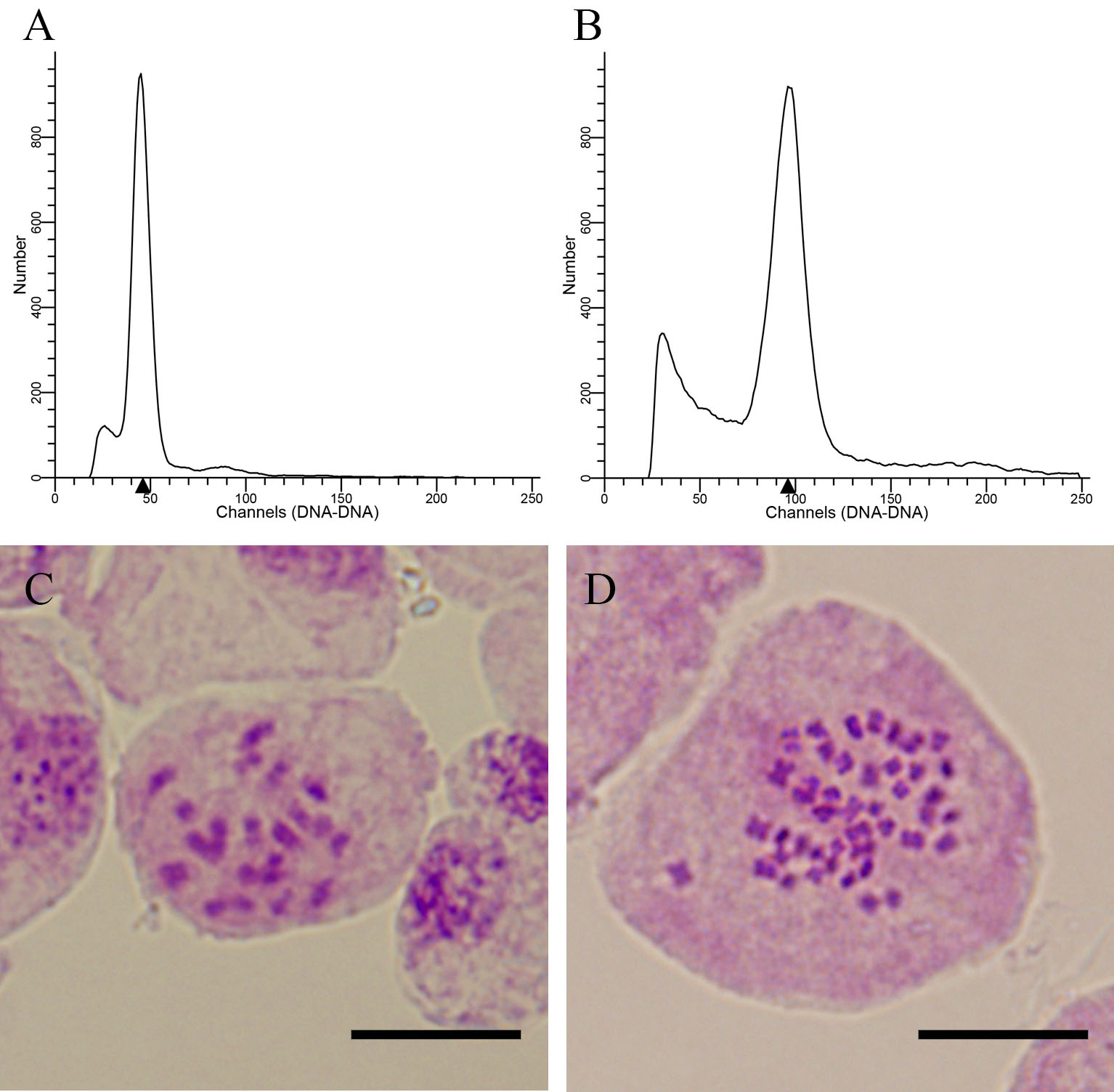

Supplement: Supplementary file 1 [file Data_Sheet_2.ZIP › Fig 2.jpg]

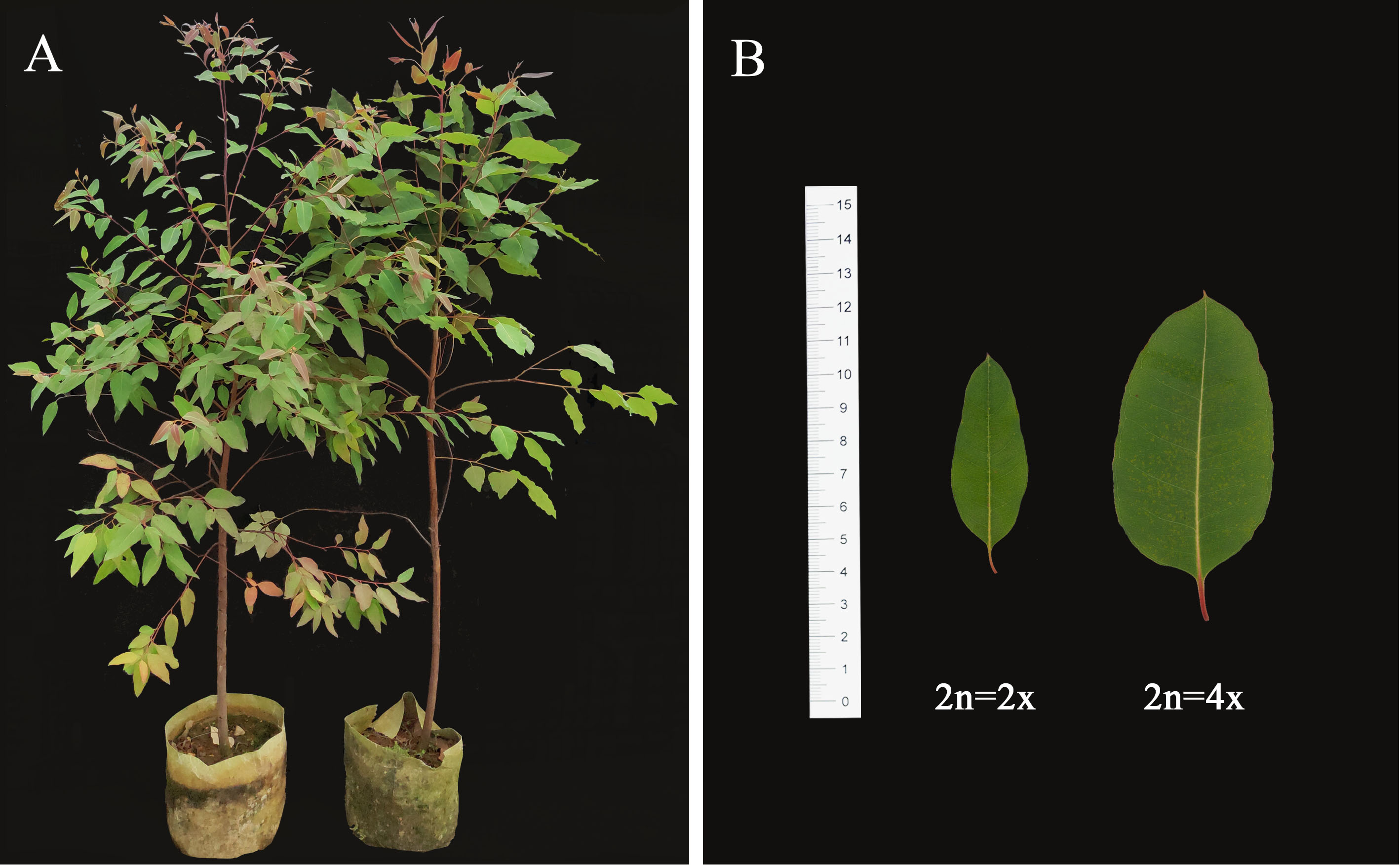

Supplement: Supplementary file 1 [file Data_Sheet_2.ZIP › Fig 3.jpg]

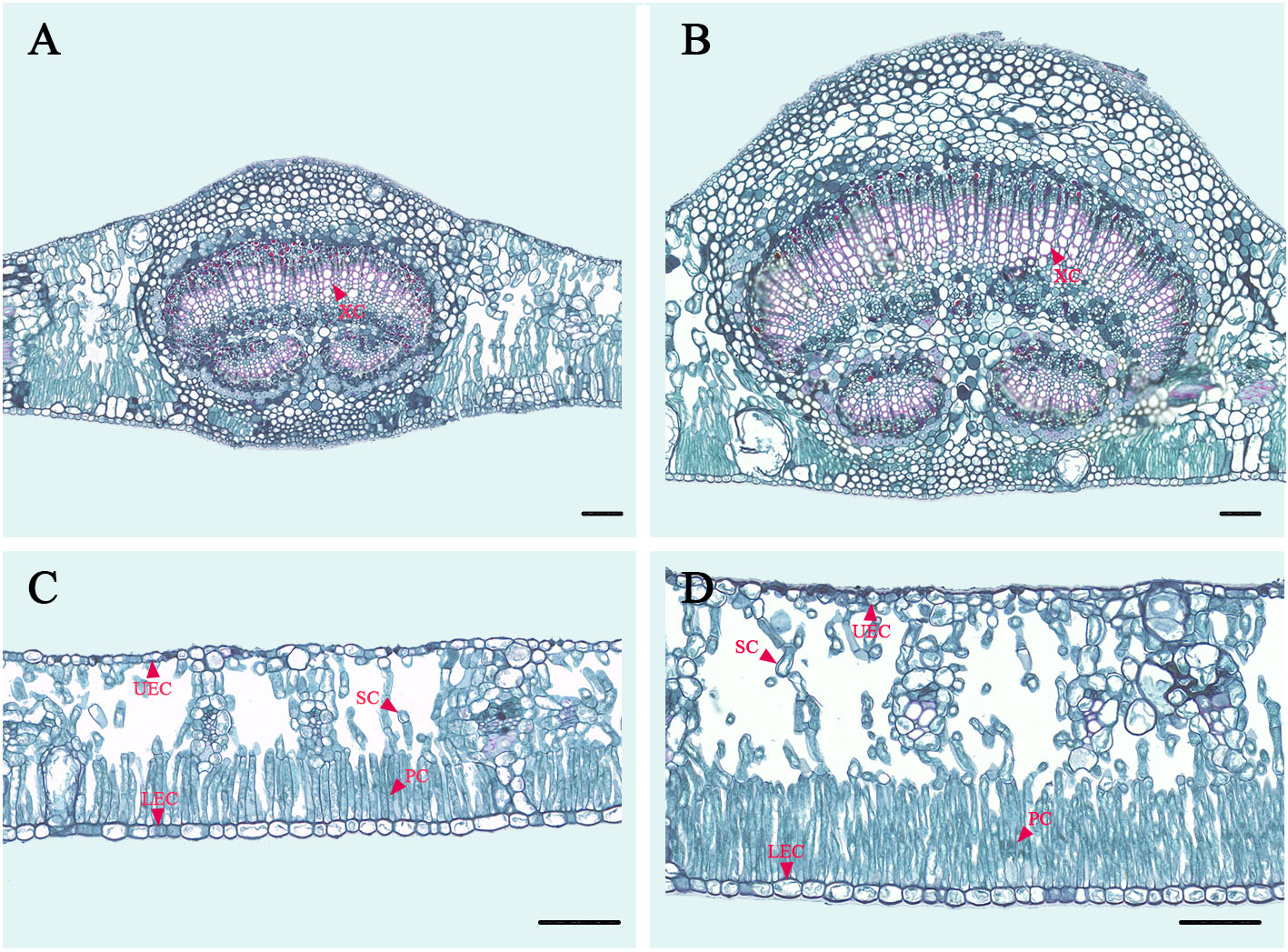

Supplement: Supplementary file 1 [file Data_Sheet_2.ZIP › Fig 4-revised.jpg]

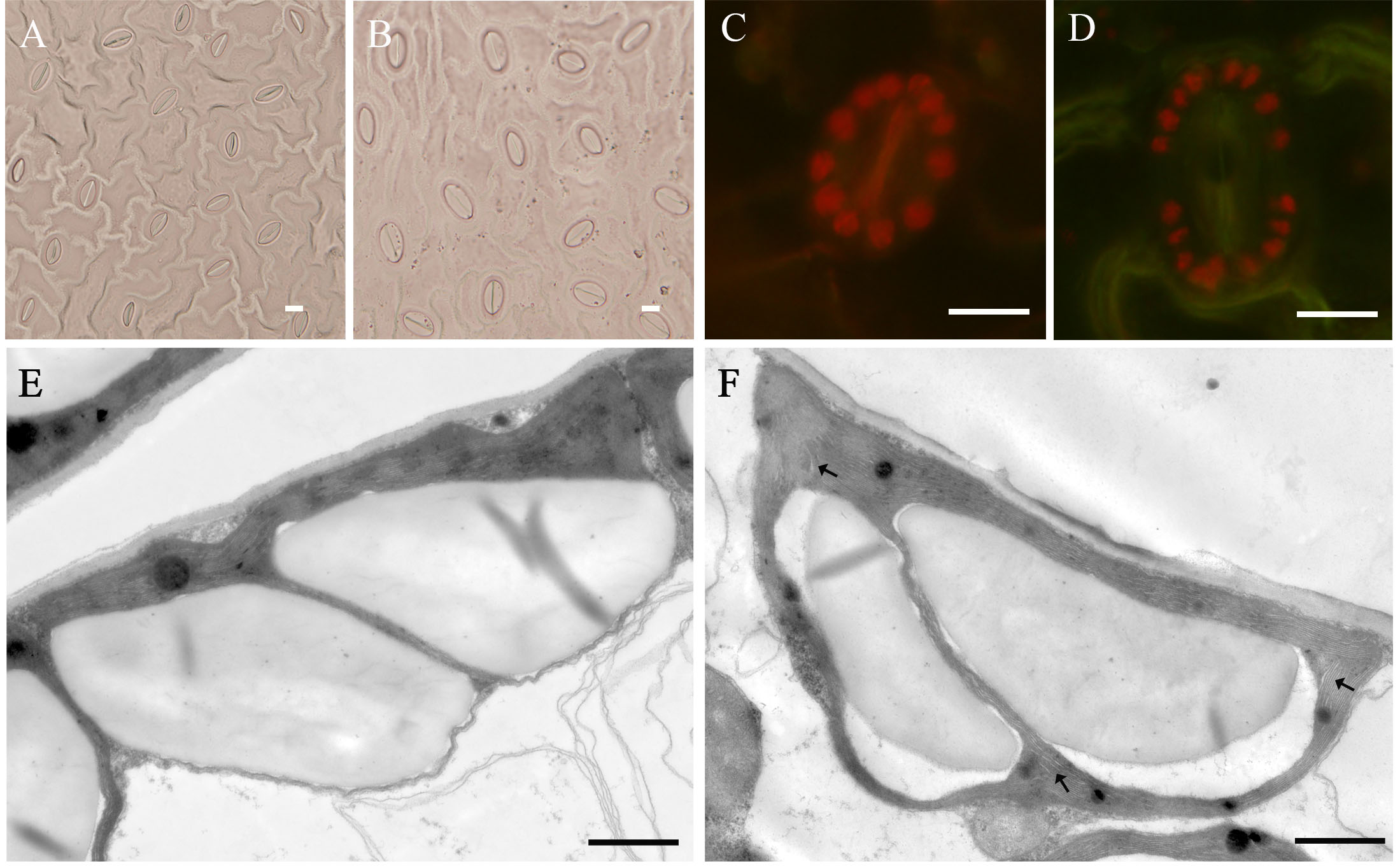

Supplement: Supplementary file 1 [file Data_Sheet_2.ZIP › Fig 5.jpg]

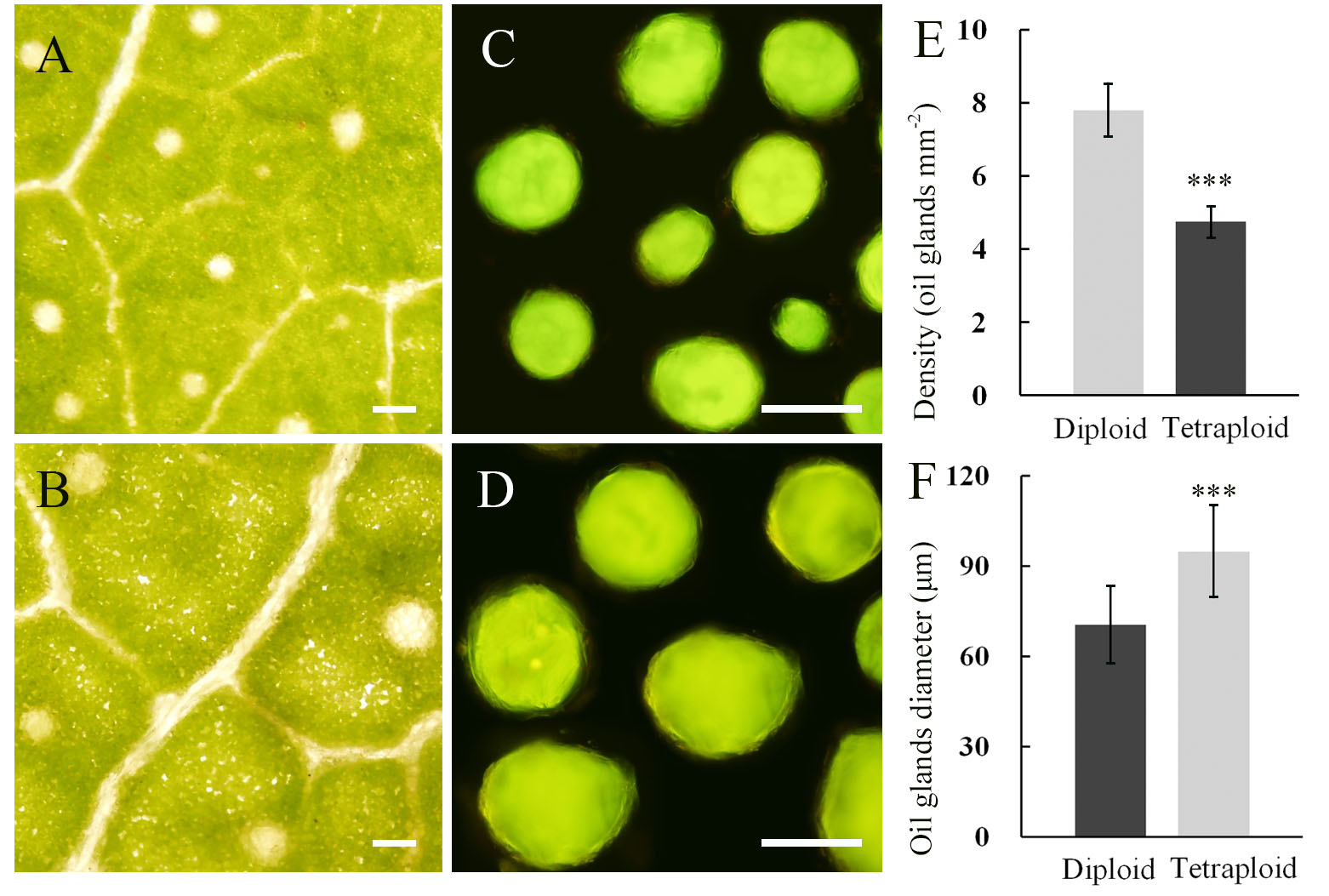

Supplement: Supplementary file 1 [file Data_Sheet_2.ZIP › Fig 6.jpg]
